# When Digital Connectivity Drivers Meet Digital Disconnection: A Cross-Country Study on Smartphone Checking, Digital Disconnection Strategies, and Digital Stress

**DOI:** 10.1177/20501579251378561

**Published:** 2025-09-29

**Authors:** Kevin Koban

**Affiliations:** 1 Department of Communication, 27258 University of Vienna, Vienna, Austria

**Keywords:** Cross-sectional survey, cross-country survey, smartphone habits, digital disconnection, digital stress

## Abstract

In today's digitized societies, where constant connectivity is perceived as a social norm, many people voluntarily seek temporary digital disconnection to balance out benefits and harms. Given that any direct effectiveness of digital disconnection is limited, recent approaches emphasized its conditionality. Conducted in two culturally dissimilar countries (the United States and Indonesia), the present large-scale cross-sectional youth surveys (*N*s = 1,922 and 2,107, respectively) explored whether different popular feature- and rules-based digital disconnection strategies (digital detox app use vs. reflective smartphone disengagement vs. digital solitude) may serve as an ambivalent moderator for relationships between habitual and compulsive smartphone checking, on one side, and digital stress experiences, on the other. For U.S. youth only, results indeed suggested that reflective smartphone disengagement might minimize detrimental side effects of firm checking habits, whereas it exacerbates links to digital stress for people who struggle with smartphone checking compulsiveness.

Mobile social media's pervasiveness together with a perceived imperative to comply with connectivity norms in digitized societies has led to a seemingly paradoxical simultaneity of desires for both digital connection and digital disconnection (i.e., voluntary non-use of digital media) (Nassen et al., 2023). As a result, many people's lives are signified by conflicts between the benefits and costs of digital connectivity (Vanden Abeele, 2021). These conflicts are symptomatic for conflicting responsibilities toward others and oneself (Ytre-Arne et al., 2020) and so prevalent that commercialized “solutions” have established themselves (Jorge et al., 2022). However, available evidence about the effectiveness of such digital detox solutions is mixed (Ansari et al., 2024).

According to Vanden Abeele et al. (2024), it is indeed essential that digital disconnection is not understood as a universally effective silver bullet but instead as practices where *some strategies* might work *indirectly rather than directly* for *some people* under *certain conditions*. Bearing in mind that digital disconnection scholarship is still solidifying, conceptually nuanced research exploring contingencies is needed.

In this spirit, this study investigates the simultaneity of internalized drivers of digital connection and engagement in digital disconnection via two cross-sectional surveys of emerging adults (i.e., 16–25-year-olds) (Arnett, 2007) from an individualistic and a collectivistic country (i.e., the United States and Indonesia, respectively). Specifically, it is explored how digital detox app use (as a feature-based strategy), reflective smartphone disengagement, and digital solitude (as distinct rule-based strategies) moderate links between habitual and compulsive smartphone checking (as two related, yet conceptually distinct, connectivity drivers) and digital stress. Following Steele et al. (2020) and Hall et al. (2021), digital stress is conceptualized as a comprehensive construct capturing mentally overburdening psychological consequences associated with (almost) permanent connectivity that involves five facets: availability stress, approval anxiety, fear of missing out (FOMO), connection overload, and online vigilance. Shortly put: This study aims for examining the what (i.e., what disconnection strategy), when (i.e., accounting for habitual and compulsive smartphone checking tendencies), and for whom (i.e., U.S. and Indonesian youth) of how digital disconnection might work, thus contributing to a young field with limited nuanced evidence.

## Habitual and Compulsive Smartphone Checking as Gateway Enlargements

Most people tend to build mental representations of repeatedly performed and rewarded everyday life behaviors that, when cued, are automatically activated and also executed unless inhibitory control intervenes (Wood & Rünger, 2016), including media technology-related behaviors (Bayer & LaRose, 2018). Smartphone checking is considered a prime example of a habitualized behavior that has been scrutinized as a gateway toward psychosocially harmful or even addictive smartphone activities (Oulasvirta et al., 2012).

It needs to be emphasized, though, that habitual smartphone checking is not inherently dysfunctional nor should it be understood as a clear sign of problematic addictive tendencies (Aagaard, 2021). On the contrary, mobile media habits are the consequence of a well-functioning mental architecture aiming for processual efficiency of goal-directed behavior (e.g., ubiquitous informational, social, or entertainment rewards related to mobile social media) (Naab & Schnauber, 2016) simply by reducing users’ investment of mental resources into cognitively costly self-observation. Given that habits can also be instigated when initial rewards are weakened or even absent and that people's goals are rarely unitary, goal-incongruent smartphone habits are likely to persist even if costs (slightly) outweigh benefits (i.e., “bad” habits) (Wood & Neal, 2007). In most cases, however, habit execution can be inhibited with intentions to do so (Gardner et al., 2020).

Given that habitual smartphone checking, therefore, merely refers to downsized self-observation that is reflected by high levels of automaticity, lacking awareness, and cognitive efficiency, it needs to be distinguished from compulsive smartphone checking, which, in turn, represents a profound lack of controllability through (chronically) diminished inhibitory self-reaction (LaRose, 2010). Accordingly, unlike habits where responses are automatically prepared but not necessarily initiated (Hardwick et al., 2019), compulsive smartphone checking can be characterized by an irresistible urge that cannot be inhibited even under extremely costly circumstances (Clements & Boyle, 2018), making it more likely to be detrimental across contexts (Seo & Ray, 2019). In other words, while habits represent a learned reduction of intentionally exercised self-observation and schema-activation self-control, compulsion refers to an inability to stop a certain behavior from being executed. Or more specifically, habitual smartphone checking is primarily about reduced self-control over checking one's smartphone when cued, whereas compulsive smartphone checking points toward reduced self-controllability over it (regardless of whether intention is underlying or not).

Extant research suggests that amplified smartphone checking is indicative of goal conflicts underlying digital stress (Bayer et al., 2016). Accordingly, both habitual and compulsive checking may be characterized as gateway enlargements associated with digital stress. Meier (2022) and Meier et al. (2023) indeed reported positive links between smartphone checking habits and daily life interruptions and task delay, respectively. However, others, accounting for conceptual overlap, only found meaningful relationships with information and communication overload for compulsive, not for habitual, checking (Koban et al., 2023). Compulsive smartphone checking, and compulsive social media behavior in general, have further been linked consistently to other digital stress facets like availability stress (Li & Chan, 2024) or FOMO (Dhir et al., 2018).

In sum, there is solid conceptual and some empirical reason to assume distinct relationships with digital stress for habitual and compulsive smartphone checking. While habits appear to be inconclusively located between gateway enlargement (suggesting a positive relationship) and qualifications thereof (via cognitive efficiency and behavioral inhibition), compulsion points toward overwhelming available cognitive and motivational resources for dealing with connectivity demands.

**RQ1:** How is habitual smartphone checking related to digital stress?
**H1:** Compulsive smartphone checking is positively related to digital stress.
**H2:** Compulsive smartphone checking exhibits a greater positive relationship with digital stress than habitual smartphone checking.

## Digital Disconnection Strategies: Many Shapes, Questionable Effectiveness

Digital disconnection comes in many shapes, varying, for instance, in terms of permanence (e.g., taking brief non-use-breaks vs. quitting completely), rigorousness (e.g., slight reduction vs. full abstinence), comprehensiveness (e.g., limited to a single vs. covering all available devices, platforms, features, etc.), or its primary mode (e.g., by limiting time, access, channels, contents, contacts, and features) (Vanden Abeele et al., 2024). Conceptually, digital disconnection can be differentiated between feature-based (e.g., using online and offline tools for obscuring, disabling, or removing connection possibilities) and rule-based (e.g., setting up temporal, locative, or situational boundaries) disconnection strategies (Nguyen et al., 2022). What all of these strategies have in common is that they are meant to control lacking, reclaim temporarily lost, or overcome challenges to users’ agency over digital connectivity and related psychological harms (Vanden Abeele et al., 2022).

Although a recent meta-analysis identified small overall benefits (Ansari et al., 2024), existing evidence on the effectiveness of digital disconnection across strategies and outcomes is altogether mixed (Nassen et al., 2023). Although many users tend to appreciate voluntary non-use (Nguyen, 2021), they are typically aware of social (e.g., loss of geographically distant connections, FOMO, social disapproval), practical (e.g., loss of convenient information gathering, loss of boredom relief and entertainment), and participatory (e.g., loss of civic participation) challenges that lead them to balance out connectivity and disconnectivity efforts. In cross-sectional surveys, it is further virtually impossible to determine cause and effect, meaning that, for instance, positive correlations could either suggest harmful consequences related to digital disconnection or a sense of urgency under which they have been engaged. Overall, it nevertheless appears as if highly disruptive strategies are most likely impractical and detrimental in digitized societies, whereas moderate approaches where users are nudged to be more agentic may be a favorable compromise (Karsay & Vandenbosch, 2021).

Moderate feature-based disconnection typically involves health apps that, among other functions, allow users to self-track their digital activities, set goals for desired non-use, or block notifications, alerts, calls, or temporarily even the whole device (Rahmillah et al., 2023), thus essentially reducing the chance for potential triggers of digital stress (e.g., seeing peers being available or visibly judging others’ posts, noticing others’ socially rewarding experiences, receiving several notifications at once) to occur or, at least, for someone to become aware of them. Previous research has demonstrated that engaging such apps can prevent heavy social media use from turning problematic (Schmuck, 2020) or increase self-efficacy toward goal-directed behavior (Keller et al., 2021).

Concerning rule-based disconnection strategies, there is a great variety of, sometimes highly idiosyncratic, behaviors (Nguyen et al., 2022). Reflective smartphone disengagement and digital solitude provide overarching conceptualizations with distinct priorities. Reflective smartphone disengagement (defined as “deliberatively develop[ed] rules for when and how it is appropriate to use the smartphone”; Matthes et al., 2022, p. 3) primarily focuses on controlling and reducing the perceived role of smartphones in one's daily life, highlighting specific situations where they may be inappropriate. Aside from situationally minimizing contact with potentially triggering stimuli, such deliberately self-imposed rules may, even more so than feature-based strategies, reflect a way to regain agency over one's digital well-being (Karsay & Vandenbosch, 2021) or, more generally, a cross-situational inclination toward an agentic mindset concerning mobile connectivity (Hefner & Freytag, 2024). Said differently: Engaging in reflective smartphone disengagement, directly or via stance, could help young people to not let others’ actual (or imagined) expectations about constant availability, potentially rejecting responses to one’ posts, missed out opportunities for social rewards, incoming amounts of digital information, or supposed significance of always having one's phone at hand be perceived as overwhelming and thus stressful. Available evidence indeed indicates reflective smartphone disengagement's over-time benefits across digital stress phenomena (e.g., information overload, perceived availability norms, nomophobia, FOMO) and also general health indicators (e.g., feelings of loneliness) (Matthes et al., 2022, 2023).

Solitude has recently been reconceptualized for hybrid social realities as noncommunication (Campbell & Ross, 2022) or unavailability to communicate with others (Ross et al., 2023). Ergo, digital solitude can be conceptualized similarly, but only covering digital media, thus more strongly tapping into what Klingelhoefer et al. (2024) documented to be a primary motivation for digital disconnection: to be present offline. Recent findings on voluntary solitude point toward it being beneficial for refueling individuals’ social batteries (Ross et al., 2023), which arguably is at the heart of many digital stress experiences (most notably, availability stress, approval anxiety, FOMO, and connection overload).

However, based on the recently theorized contingent nature of disconnectivity effects, mixed available evidence on effectiveness, and methodological shortcomings of cross-sectional surveys, it nonetheless seems unreasonable to formulate a concrete hypothesis about the direction of how engagement in digital disconnection is related to digital stress or whether different strategies differ in this regard.

**RQ2:** How does engagement in digital disconnection (i.e., digital detox app use, reflective smartphone disengagement, and digital solitude) relate to digital stress?

## Beneficial and Harmful Simultaneity of Digital Connectivity and Disconnectivity

Aside from social, practical, and participatory challenges, connectivity drivers like habitual or compulsive checking could further alleviate disconnection effectiveness or even end up making it harmful. Relying on willpower to disconnect is notoriously ambitious, given how demanding it is to (successfully) exercise self-control (Brevers & Turel, 2019) and possible negative consequences of resource depletion (Berger et al., 2018).

Instead of counting on self-control resources, digital detox apps and also rule-based approaches favor preventing contextual cues from activation. Such contextual discontinuity can take various forms, for instance, by limiting the salience of technical cues such as notifications or by avoiding mere smartphone visibility (Bayer et al., 2016).

For habits, contextual discontinuity has often been regarded as highly promising for behavioral change because it introduces friction to an otherwise purposely frictionless experience, leading to more mindful and intentional actions (Verplanken & Orbell, 2022). Temporary contextual discontinuity through moderate digital disconnection strategies may therefore reduce the likelihood that automatically instigated smartphone checking occurs, offering a break from being permanently confronted with potential digital stress-inducing stimuli without risking adverse psychosocial reactions or any meaningful loss of connectivity benefits (in contrast to Dekker et al., 2025, where a more rigorous disconnection strategy was tested). It might maximize the goal-congruent basis of habitual smartphone checking while minimizing goal-incongruent side effects of an enlarged gateway.

**H3:** Engagement in digital disconnection (i.e., digital detox app use, reflective smartphone disengagement, and digital solitude) is a negative (i.e., inhibiting) moderator for how habitual smartphone checking is related to digital stress.

For compulsive smartphone checking, on the other hand, contextual discontinuity might come with heightened costs. A temporary loss of opportunity to satisfy one's urge for checking one's smartphone can result in intense craving during digital disconnection episodes (i.e., amplifying internalized digital stressors) and (over-)compensating relapse afterwards (i.e., increased likelihood of being overwhelmed). Preliminary evidence concerning such counterproductive consequences of digital disconnection can be found in studies where notifications were temporarily disabled. Here, participants consistently experienced increased levels of stress, anxiety, and FOMO, and responded by seeking more connectivity compared to when notifications were activated normally, particularly for those exhibiting trait-level FOMO (Dekker et al., 2025). While it was not explicitly tested, it appears plausible that such detrimental responses are more likely for users with compulsive checking tendencies, and also that it can be translated to digital stress.

**H4:** Engagement in digital disconnection (i.e., digital detox app use, reflective smartphone disengagement, and digital solitude) is a positive (i.e., intensifying) moderator for how compulsive smartphone checking is correlated with digital stress.

## Cross-Country Meanings of Digital Connectivity and Disconnectivity

Although digital disconnection research has been thriving in recent years, it has also been noted that it focuses primarily on Western, educated, industrialized, rich, democratic societies, underestimating the role of varying political, societal, and infrastructural conditions (Nguyen & Hargittai, 2023). Even when comparing Global North and Global South societies with similar mobile social media penetration, cross-country differences concerning, for example, shared individualist or collectivist values (i.e., socially reinforced emphases on independence and individual autonomy or interdependence and communal cohesion, respectively) (Hofstede, 2011) can have a profound impact on whether and how digital disconnection is practiced, complicated, and linked to digital stress. While digital disconnection may be typically understood as self-empowerment resulting from individual responsibility for one's own digital well-being in individualist cultures (Jorge et al., 2022), collectivist cultures’ tendency toward interdependence may prioritize relationship maintenance, such that social consequences become more salient (Ross et al., 2023). Conducting the present study in an individualistic Global North and a collectivistic Global South country may therefore provide additional exploratory insights into cross-country meanings of digital connectivity and disconnectivity.

**RQ3:** How do associations between engagement in habitual and compulsive smartphone checking, digital disconnection (i.e., digital detox app use, reflective smartphone disengagement, and digital solitude), and digital stress differ across samples from an individualist and a collectivist society?

## Method

The study was conducted as part of a comprehensive survey about young people's social media use in the United States and Indonesia. The project was screened by the internal review board to be of minimal risk. Aside from demographics, reflective smartphone disengagement has already been used in another project where it served a different role (i.e., as an outcome) to answer distinct research questions. Supplementary Material, including scale wordings in both languages, anonymized datasets, scripts, and outcome files, is available at https://osf.io/mq68b/?view_only=7f38ed3753bd488cb432c3612ef0d799.

### Participants

Surveys involving emerging adults between 16 and 25 years were conducted in the United States (August 2022) and Indonesia (December 2022). Recruitments targeted equal distributions concerning both gender identification and age (i.e., half being between 16 and 20, half being between 21 and 25 years old). Social media users who (a) provided active informed consent, (b) completed the full survey, and (c) did not identify as a non-binary gender (given that *N* = 68 was deemed too small for meaningful results) were included. Participants who (d) failed attention checks or (e) speeded through the survey were also excluded. The final samples included *N* = 1,922 (age *M* = 21.26; *SD* = 2.52; *n* = 937 females) and *N* = 2,107 (age *M* = 20.89; *SD* = 2.37; *n* = 1,048 as female) participants from the United States and Indonesia, respectively (see OSF Supplementary Table 2(a) for additional demographics).

### Procedure

After providing informed consent, participants were asked for sociodemographic information and general social media use, followed, in randomized order, by item blocks about (a) mental health influencers, (b) habitual and compulsive smartphone checking, (c) social media monitoring and peer experiences, (d) digital stress, (e) dispositional traits, (f) digital disconnection, (g) psychological well-being, and (h) experiences with mental health issues. After that, another block about (i) experiences with cyberbullying and hostile social media content was presented. Scales within blocks and items within scales were randomly ordered.

### Measures

Given that the initial survey was conducted in English, a translation back-translation procedure was applied for the Indonesian survey with two native Indonesians with proficient English skills. Afterward, translators resolved disagreements to improve clarity through discussion. A full item list with scale instructions is provided in OSF Supplementary Table 1(a)–(c). Unless specified otherwise, 5-point Likert scales were used. Except for reflective smartphone disengagement and digital stress, existing items and scale instructions were slightly adapted.

#### Habitual and Compulsive Smartphone Checking

Habitual and compulsive smartphone checking was measured, respectively, via a four-item version of the non-specific self-report habit index (Gardner et al., 2016), which primarily focuses on capturing automaticity and lack of awareness (as pivotal definitional criteria of habits), and four items adapted from Seo and Ray (2019), which predominantly address a sense of urgency and lacking controllability (as key characteristics of compulsion). Confirmatory factor analyses (CFAs) demonstrated good global fit for a two-factor solution in both the United States, χ^2^(19) = 102.76, *p* < .001, CFI = .985, RMSEA = .048 (90%-CI[.040;.056]), SRMR = .031, and the Indonesian sample, χ^2^(19) = 70.72, *p* < .001, CFI = .991, RMSEA = .036 (90%-CI[.029;.043]), SRMR = .020. Latent factor cross-correlation indicated a substantial amount of shared variance, however, without pointing to conceptual redundancy (*r*s = .658–.676). Measurement invariance testing further displayed partial metric invariance across languages after loosening the equality constraint for a single item, Δχ^2^(5) = 10.67, *p* = .058. Across samples, reliability was good for habitual and compulsive smartphone checking (see OSF Supplementary Table 2(b)). Notably, U.S. youth scored higher on habitual smartphone checking, *t*(3920.2) = 2.46, *p* = .014, Cohen's *d* = .08, while Indonesian participants did so on compulsive smartphone checking, *t*(3939.6) = −12.66, *p* < .001, *d* = −.40.

#### Digital Disconnection

In order to cover digital disconnection along existing conceptual spectra, three practices were operationalized. First, participants’ frequency of using *digital detox apps* during the past four weeks was captured with three items from Schmuck (2020), after providing them with a brief description to standardize understandings. Second, *reflective smartphone disengagement* was measured via a 6-item scale provided by Matthes et al. (2022), in which participants indicated whether they deliberatively follow situational rules about their smartphone use. Third, four items from Palgi et al.'s (2021 ) positive solitude scale were adapted to address *digital solitude* as non-compulsory digital unavailability (Campbell & Ross, 2022). Specifically, participants specified their motivation to be offline.

CFAs revealed good global model fit for a three-factor solution in both the U.S. sample, χ^2^(62) = 316.82, *p* < .001, CFI = .967, RMSEA = .046 (90%-CI[.042;.051]), SRMR = .039, and the Indonesian sample, χ^2^(62) = 174.47, *p* < .001, CFI = .985, RMSEA = .029 (90%-CI[.025;.034]), SRMR = .027. While reflective smartphone disengagement and digital solitude correlated strongly in both samples (*r*s = .677–.694), this shared variance may not be enough to point toward redundant constructs. Testing for measurement invariance provided evidence for partial metric invariance after releasing a single digital solitude indicator from equality constraints, Δχ^2^(9) = 13.04, *p* = .161. Across samples, reliability was good for digital detox app use and digital solitude, and acceptable for reflective smartphone disengagement (see OSF Supplementary Table 2(b)). Welch's *t*-tests showed that Indonesians scored significantly higher across digital detox app use, *t*(3917.5) = −11.40, *p* < .001, *d* = −.36, reflective smartphone disengagement, *t*(3816.8) = −19.57, *p* < .001, *d* = −.62, and digital solitude, *t*(3773.0) = −12.73, *p* < .001, *d* = −.40.

#### Digital Stress

Digital stress was assessed with the eponymous scale by Hall et al. (2021), including availability stress (four items), approval anxiety (six items), fear of missing out (four items), connection overload (six items), and online vigilance (four items), where participants indicated how frequently they felt like the described scenarios over the past 4 weeks. Second-order CFAs with each sub-phenomenon being entered as first-order constructs showed good global fit in both the U.S. sample, χ^2^(247) = 1125.96, *p* < .001, CFI = .956, RMSEA = .043 (90%-CI[.041;.045]), SRMR = .045, and the Indonesian sample, χ^2^(247) = 1624.81, *p* < .001, CFI = .941, RMSEA = .051 (90%-CI[.049;.054]), SRMR = .056. Measurement invariance testing indicated partial metric invariance after releasing equality constraints for nine first- and one second-order indicator, Δχ^2^(13) = 22.02, *p* = .055. Across the U.S. and Indonesian samples, reliability coefficients were good for availability stress, fear of missing out, and online vigilance, good-to-excellent for connection overload, and excellent for approval anxiety, as well as good for higher-order digital stress (see OSF Supplementary Table 2(b)). On average, Indonesian participants scored higher on availability stress, *t*(3917.6) = −12.00, *p* < .001, *d* = −.38, approval anxiety, *t*(3981.3) = −8.53, *p* < .001, *d* = −.27, connection overload, *t*(3920.8) = −4.56, *p* < .001, *d* = −.14, and online vigilance, *t*(3838.1) = −9.89, *p* < .001, *d* = −.31, but not on fear of missing out, *t*(4012.6) = .023, *p* = .819, *d* = .01. Overall, Indonesians also had significantly higher scores for digital stress, *t*(3879.1) = −8.54, *p* < .001, *d* = −.27.

#### Covariates

Gender and education were measured via closed-ended items, age via slider scale. Participants were provided with several options to cover educational backgrounds in either country, which were collapsed into presence or absence of post-secondary education.

### Statistical Analysis

A multi-group path analysis was conducted using maximum likelihood estimation with robust standard errors. All variables included in interaction terms were mean-centered. Path coefficients were compared via defined contrasts and via nested-model comparison using Akaike information criterion (AIC) and Bayesian information criterion (BIC), which have varying properties and penalize model specifications differently (Vrieze, 2012).

## Results

**Figure 1.**
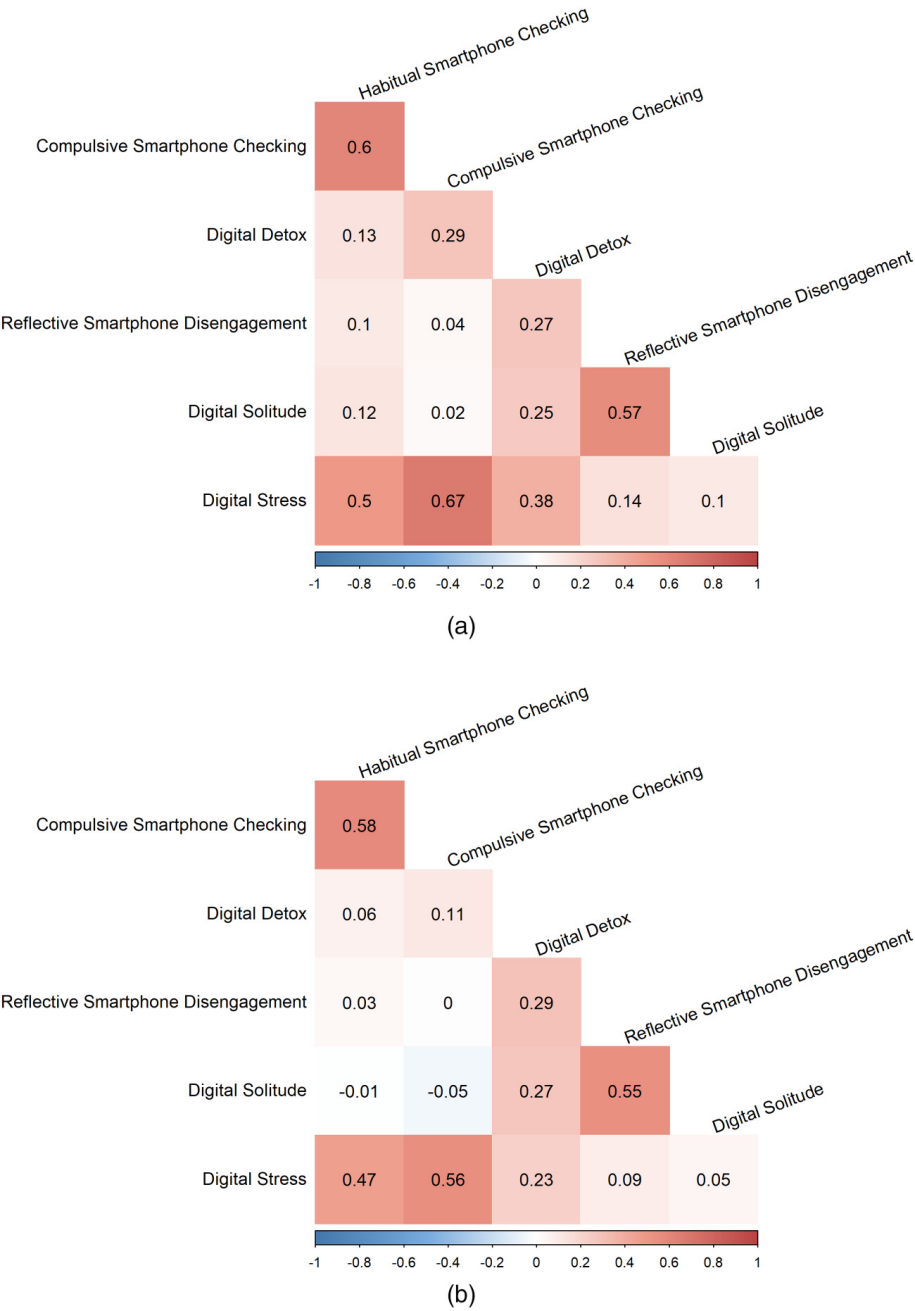
(a) Zero-Order Correlations for the U.S. Sample. (b) Zero-Order Correlations for the Indonesian Sample.

Zero-order correlations are presented in Figure 1(a) and (b). Table 1 displays main results. Exploratory analyses where each digital stress subdimension was analyzed separately can be found on OSF (see OSF Supplementary Output Files titled “Exploratory Analysis” and “Exploratory Analysis: Contrasts,” as well as OSF Supplementary Tables 3 and 4(a)–(c)).

**Table 1.** Results From the Path Models.

	U.S. sample (*N* = 1,922)		Indonesian sample (*N* = 2,017)	
	*b* (*SE*), β	*p*	*b* (*SE*), β	*p*
Habitual smartphone checking	.13 (.02), .15	<.001	.16 (.02), .20	<.001
Compulsive smartphone checking	.45 (.02), .53^a^	<.001	.32 (.02), .39^a^	<.001
Digital detox app use	.15 (.02), .20	<.001	.11 (.01), .15	<.001
Reflective smartphone disengagement	.09 (.03), .09	<.001	.03 (.03), .02	.359
Digital solitude	−.02 (.02), −.02	.419	.01 (.02), .01	.682
Gender (1 = male, 2 = female)	.001 (.03), .001	.973	.04 (.03), .02	.173
Age	−.01 (.01), −.03	.090	−.02 (.01), −.06	.010
Educational background (1 = no university degree, 2 = university degree)	.09 (.04), .04	.013	.05 (.04), .03	.163
Habitual smartphone checking × digital detox app use	.002 (.02), .004	.879	−.01 (.02), −.02	.532
Compulsive smartphone checking × digital detox app use	.01 (.02), .02	.389	.02 (.02), .02	.372
Habitual smartphone checking × reflective smartphone disengagement	−.04 (.02), −.06^a^	.045	.04 (.03), .05^a^	.143
Compulsive smartphone checking × reflective smartphone disengagement	.05 (.02), .06	.038	.05 (.03), .05	.099
Habitual smartphone checking × digital solitude	.02 (.02), .03	.316	.01 (.02), .02	.576
Compulsive smartphone checking × digital solitude	.01 (.02), .02	.520	.001 (.02), .002	.957
*R* ^2^	.516		.384	

*Note*: ^a^ indicates significant country differences.

### U.S. Sample

Path analysis resulted in significant positive correlations with digital stress for both habitual, β = .15, *p* < .001 (answering RQ1), and compulsive smartphone checking, β = .53, *p* < .001 (supporting H1). Both nested-model comparison (ΔAIC: −86.7; ΔBIC: −25.8) and defined contrast (Δ*b* = .33, *SE* = .04, *p* < .001) indicated that links with digital stress are more pronounced for compulsive than habitual smartphone checking, which supports H2.

Results revealed significant positive correlations with digital stress for digital detox, β = .20, *p* < .001, and reflective smartphone disengagement (hereinafter, in Results, called RSD), β = .09, *p* < .001, but not for digital solitude, β = −.02, *p* = .419. Ergo, greater use of digital detox apps and situationally self-restrictive smartphone use were related to more frequent digital stress experiences (answering RQ2). Pair-wise nested-model comparisons and defined contrasts showed solid evidence for differences between digital detox and digital solitude (ΔAIC: −36.8; ΔBIC: −31.2; Δ*b* = .17, *SE* = .03, *p* < .001), and inconsistent differences for RSD and digital solitude (ΔAIC: −2.1; ΔBIC: 3.5; Δ*b* = .11, *SE* = .04, *p* = .008), and digital detox and RSD (ΔAIC: −6.0; ΔBIC: −0.4; Δ*b* = .06, *SE* = .03, *p* = .062).

**Figure 2.**
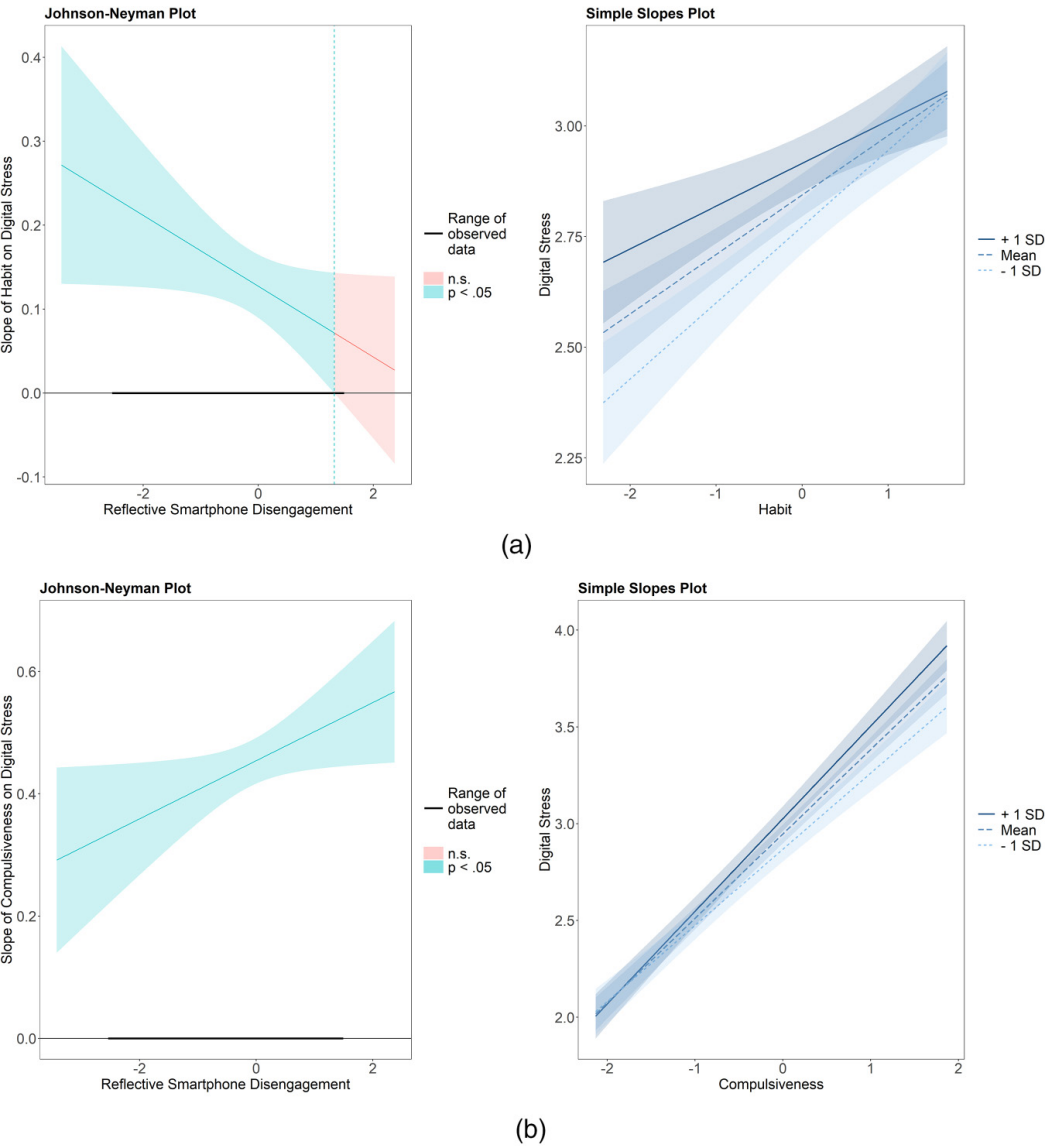
(a) Johnson–Neyman and Simple Slopes Plots of the Significant Interaction Effect Between Reflective Smartphone Disengagement and Habitual Smartphone Checking on Digital Stress. (b) Johnson–Neyman and Simple Slopes Plots of the Significant Interaction Effect Between Reflective Smartphone Disengagement and Compulsive Smartphone Checking on Digital Stress.

RSD emerged as significant moderator for both habitual, β = −.06, *p* = .045, and compulsive checking, β = .06, *p* = .038. As shown in Figure 2(a) and (b), greater scores on RSD came with weaker associations between habitual checking and digital stress, while it was related to stronger links between compulsive checking and digital stress. No interaction effect with digital detox or digital solitude turned out significant. Thus, H3 and H4 were only partially supported.

### Indonesia Sample

Results revealed significant positive correlations with digital stress for both habitual, β = .20, *p* < .001 (answering RQ1), and compulsive smartphone checking, β = .39, *p* < .001 (supporting H1). Both nested-model comparison (ΔAIC: −18.1; ΔBIC: −12.4) and defined contrasts (Δ*b* = .16, *SE* = .04, *p* < .001) suggested that associations with digital stress are stronger for compulsive than habitual smartphone checking, again providing support for H2.

Path analysis also demonstrated a significant positive correlation with digital stress for digital detox, β = .15, *p* < .001, but not for RSD, β = .02, *p* = .359, nor digital solitude, β = .01, *p* = .682. That is, only greater use of digital detox apps was related to more frequent digital stress experiences (answering RQ2). In pair-wise nested-model comparisons and defined contrasts, solid evidence emerged for differences between digital detox and digital solitude (ΔAIC: −11.2; ΔBIC: −5.5; Δ*b* = .10, *SE* = .03, *p* < .001), inconsistent evidence for differences between digital detox and RSD (ΔAIC: −5.7; ΔBIC: 0; Δ*b* = .08, *SE* = .03, *p* = .012), and no evidence at all for differences between RSD and digital solitude (ΔAIC: 1.8; ΔBIC: 7.5; Δ*b* = .02, *SE* = .04, *p* = .716).

No significant interaction effects between either habitual or compulsive checking and any form of digital disconnection could be found, meaning H3 and H4 were not supported.

### Country Comparison

Examining path coefficients via pair-wise nested-model comparisons and defined contrasts suggested cross-country variance for compulsive checking predicting digital stress (ΔAIC: −22.1; ΔBIC: −15.7; Δ*b* = .14, *SE* = .03, *p* < .001), suggesting a significantly stronger positive association in the U.S. than in the Indonesian sample. Defined contrasts further revealed a significant difference for the interaction term of habitual checking and RSD, Δ*b* = −.09, *SE* = .04, *p* = .019, which turned out (significantly) negative in the U.S. but (non-significantly) positive in the Indonesian sample. Answering RQ3, these findings highlight a few country-specific tendencies; however, the scarcity of country variation more conclusively indicates broad invariance.

## Discussion

Internalized drivers of digital connection and simultaneously present digital disconnection efforts to deal with their psychosocial aftermath complicate reaching digital well-being (Vanden Abeele, 2021). In this light, this study explores contingencies of how digital disconnection does and can work (Vanden Abeele et al., 2024), arguing that habit’s plausible-yet-uncertain and compulsion's much more predictable association with digital stress might shift in different directions when moderate digital disconnection strategies, with their unique realizations of contextual discontinuity, are engaged at the same time. Results revealed significantly varying, yet nevertheless positive, relationships between both habitual and compulsive smartphone checking and digital stress experiences across country contexts that were moderated as hypothesized (i.e., inhibiting for habitual and intensifying for compulsive smartphone checking) by reflective smartphone disengagement (but neither digital detox app use nor digital solitude) in the U.S. (but not the Indonesian) youth sample. Further testing of cross-country differences for significance revealed consistency across all but a few paths, indicating, despite profound cultural and societal country differences, substantial robustness that may point to a somewhat universal smartphone culture among young people across the world (Kang & Jung, 2014).

### Compulsiveness Trumps Habits

In both samples, results indicated that firmer habitual and thoroughly compulsive smartphone checking coincides with digital stress. Importantly, links with compulsiveness exceeded those of mere habits in both countries, albeit significantly less so for Indonesian youth. Broadly, these patterns also emerged for when each digital stress dimension was analyzed separately (see OSF Supplementary Table 4(a)–(c)). On an abstract level, this country variation could be explained via differently prevailing individualist and collectivist norms, influencing whether independence from or interdependence among one's community is more valued (Hofstede, 2011). Concretely, research has documented culture-specific communication practices focusing either on self-actualization or communal experiences. For instance, Kokkoris and Kühnen (2014) demonstrated distinct authentic expression ideals across young social media users from individualist and collectivist countries. Specifically, they found that German students (i.e., from a prototypical culture) favored giving both likes and dislikes, while their Chinese (i.e., collectivist) counterparts preferred only likes without dislikes in order to appear authentic. Others have further shown that people from individualist cultures tend to value low-context direct information exchange compared to people from collectivist cultures where high-context indirect communication is typically given priority (Urakami & Lim, 2021). However, acknowledging that Indonesian youth outscored U.S. youth across most digital stress subdimensions, it might not be plausible to interpret the weaker link of compulsive checking in the former via beneficial sociotechnological conditions. Instead, it may rather be that a higher digital stress baseline (possibly partially resulting from their communal orientation and related communication patterns) simply leaves less room for more substantial increases even if participants’ checking tendency gets compulsive.

At face value, the present findings add well-powered cross-country evidence to existing arguments about distinguishing between habitual and compulsive mobile social media use (Koban et al., 2023), thus working toward conceptual clarity and avoiding overpathologization (Aagaard, 2021). Despite these conceptual considerations, it needs to be stressed that habitual and compulsive tendencies turned out to be highly correlated (i.e., *r*s = .60 and .58), indicating two crucial issues: First, it is necessity to consider what it means that both concepts are statistically “freed” from overlapping properties. In this case, it stands to question whether habitualized smartphone checking without compulsiveness and compulsive checking without automaticity are still true to their respective original meanings. Second, high correlations between conceptually distinct phenomena may point toward perceptual fuzziness. Researchers have long bemoaned constructs that are understood interchangeably by participants even though they are supposed to be distinct (Davidson et al., 2022). Turning toward participants’ understanding of items as a subject of investigation in its own right might be a worthwhile step forward for future inquiries.

### Feature-Based vs. Rule-Based Digital Disconnection

Concerning digital disconnection strategies, participants’ use of digital detox apps was related to greater digital stress in general and across most facets for both U.S. and Indonesian youth, whereas reflective smartphone disengagement was linked to more digital stress only in the U.S. sample, and selectively in terms of subdimensions at that (see OSF Supplementary Table 4(a)–(c)). Digital solitude was not significant in either sample. Comparing path coefficients revealed notable country differences: Specifically, both digital detox app use and reflective smartphone disengagement differed significantly from digital solitude in the U.S. sample, while digital detox app use did so from both reflective smartphone disengagement and digital solitude among Indonesian youth. Overall, these findings indicate that feature-based digital disconnection, and selectively also specific implementations of rule-based digital disconnection, coincide with feeling unable to cope with digital stressors.

Given the cross-sectional survey methodology, such positive associations cannot (and should not) be interpreted as decisive evidence concerning whether digital disconnection is effective or not (Vanden Abeele et al., 2024). It appears instead prudent to explicitly account for concurrence. Participants may be more likely to engage feature-based disconnection strategies when they acutely experience digital stress. Such a preference could be driven by them avoiding aversive effort involved in following self-imposed rules that need to be tailored to existing life routines, which might be easier with ready-made feature-based strategies. Beyond previously emphasized challenges for engaging in digital disconnection (Nassen et al., 2023), this interpretation would account for the notion that people can effectively choose between available disconnection strategies based on strategy-specific considerations.

What may also be plausible is that rule-based strategies are engaged on a more regular basis anyway, irrespective of whether overwhelming levels of digital stress are experienced, because they are helpful for coping with everyday mobile connectivity demands or because of cultural expectations concerning appropriateness, which is more valued in collectivist than individualist cultures (Hofstede, 2011) and might transfer to smartphone behaviors as well, thus partially explaining varying effect sizes. Comparing sample distributions, reflective smartphone disengagement and digital solitude are indeed less exceptional than digital detox app use, which might imply that the latter could be engaged once digital stress is overwhelming existing rule-based strategies. While both explanations may be speculative for now, it may be promising for future research to extend notions of interstrategic considerations and dynamics.

### Whom Digital Disconnection Might Help, and Whom it Might Hurt

Acknowledging mixed evidence both when using the higher-order digital stress construct and even more so when exploring its facets (see OSF Supplementary Table 4(a)–(c)), digital disconnection scholars recently highlighted the relevance of contingencies through which different strategies might work beneficially (Vanden Abeele et al., 2024). Innovatively situated within the contextual discontinuity literature (Verplanken & Orbell, 2022), this study argued that digital disconnection, by means of temporarily introducing friction to otherwise largely frictionless experiences, might give individuals a welcome (in case of habits) or craving-inductive (in case of compulsiveness) break from digital connectivity. Inverse interaction effects were indeed identified but only for reflective smartphone disengagement and among U.S. youth. Provided that these selective but nevertheless theoretically aligning results are not merely coincidental, this may constitute reflective smartphone disengagement as exceptionally effective for contextual discontinuation among U.S. youth, for better and for worse. For checking habits, following up on self-imposed rules for situationally inappropriate smartphone use may just provide the right balance between connectivity and disconnectivity to counter goal-incongruent routines from spiraling into progressively larger issues over time, essentially rewiring them toward their goal-congruent basis (Bayer et al., 2022). For compulsive checking, where managing one's irresistible urge might already be taxing (Brevers & Turel, 2019), reflective smartphone disengagement, apparently more so than digital detox apps and solitude, comes with increased effort needed to dynamically evaluate situational appropriateness that might trigger internalized connection cues and facilitate relapse. As such, the present findings, albeit selective, illustrate conditions under which a moderate rule-based digital disconnection strategy could maximize beneficial and minimize harmful links between digital connectivity and digital stress. Exploring reasons behind the selectivity of such interactions will be integral for digital disconnection scholarship (Vanden Abeele et al., 2024), just as it will be to further scrutinize who among young people might be more receptive to either mechanism. In this light, studying youth who identify with marginalized groups, such as the LGBTQIA+ community, whose members typically rely greatly on online spaces for identity formation (Frost et al., 2020), might be particularly valuable, provided it can be done with sufficiently powered and representative samples that involve each facet of queerness (unlike this study, where non-binary youth were excluded).

### Limitations

Several limitations need to be considered. Most obviously, cross-sectional data all but prohibits any directional or causal interpretations. Additional methodological limitations relate to use of self-reports (which are subject to response biases and possibly unsuitable for measuring firmly internationalized phenomena), closed-ended items (which lack the openness necessary for glimpsing at rich contextual information for the sake of standardization), and aggregating measurements (which cannot grasp momentary experiences that allow for within-person effect estimations). Utilizing short scales and unvalidated Indonesian translations can also be criticized given that they may come with poor measurement quality.

Concerning sampling decisions, findings from emerging adults should not be transferred to other age groups, who may differ substantially with regard to their mobile social media use. Here, general youth samples may also come with a disadvantage of overshadowing person-to-person differences and individual susceptibilities. On a similar note, U.S. and Indonesian samples were selected not only on the basis of theoretical but also pragmatic reasons due to the availability of qualified translators. While those countries can be referred to as prototypically individualist and collectivist, respectively, both cultural orientations are not homogeneous. Accordingly, premature overgeneralization toward other countries, or worse toward individualist or collectivist cultures in general, underestimates cultural, political, infrastructural, and societal heterogeneities. Then again, this study's lack of broad cross-cultural differences may be due to a global trend toward individualism (including in Indonesia) (Santos et al., 2017), which may be particularly pronounced among today's youth, who already tend toward individualist values (Beugelsdijk & Welzel, 2018), challenging whether categorizations of individualist and collectivist countries are valid.

Finally, non-binary participants were excluded based on methodological concerns (e.g., small sample size, model consistency across countries, oversimplification of unique and multifaceted experiences, potential misuse of underpowered findings). We acknowledge that such exclusions, especially if executed without sufficient reflection, can contribute to making marginalized communities less visible, which is why future research, especially on youth, may consider including additional quotas or engaging in representative community sampling.

## Conclusion

This cross-country study explored the simultaneity of firmly internalized digital connectivity drivers and practiced digital disconnection strategies regarding digital stress. Results indicate the necessity to distinguish between habitual and compulsive smartphone checking and promising avenues for better understanding feature-based and rule-based digital disconnection strategies in action. Most importantly, referring to Vanden Abeele et al.'s (2024) recently proposed digital disconnection framework, the study provides preliminary guidance concerning *what* digital disconnection strategy (i.e., reflective smartphone disengagement) might function either most beneficially or detrimentally, *when* (i.e., in case of firm habitual or compulsive smartphone checking tendencies, respectively), and for *whom* (i.e., U.S. youth). This needs to be validated using longitudinal and experimental approaches.
